# Some neighbors are more interfering: Asymmetric priming by stroke neighbors in Chinese character recognition

**DOI:** 10.3758/s13423-022-02207-9

**Published:** 2022-11-02

**Authors:** Lili Yu, Qiaoming Zhang, Meiling Ke, Yifei Han, Sachiko Kinoshita

**Affiliations:** 1 School of Psychological Sciences and Macquarie University Centre for Reading (MQCR) Macquarie University, Sydney, NSW 2109 Australia; 2 School of Educational Science, Ludong University, Yantai, Shandong China

**Keywords:** Chinese reading, Stroke neighbors, Masked priming, Character frequency

## Abstract

Chinese is a visually complex logographic script that consists of square-shaped characters, with each character composed of strokes. Previous masked priming studies using single-character Chinese stroke neighbors (i.e., visually similar characters differing in only one or two strokes, e.g., 大/犬) have shown facilitatory or inhibitory priming effects. We tested whether the mixed pattern of stroke neighbor priming might be an instance of asymmetry in priming that has been observed previously with Japanese kana and Latin alphabets. Specifically, a prime lacking a stroke (or line segment) that is present in the target speeds up the recognition of its stroke neighbor almost as much as the identity prime (e.g., 刀-刃 = 刃-刃), but not the converse (e.g., 刃-刀 >> 刀-刀). Two experiments, one using a character match task and the second using lexical decision, showed a robust asymmetry in priming by stroke neighbors. The results suggest that the early letter identification process is similar across script types, as anticipated by the Noisy Channel model, which regards the first stage of visual word recognition as a language-universal perceptual process.

## Introduction

In the last decade and a half, there has been much advance in understanding the “front-end” of reading, namely, orthographic processing – coding of letter identities and letter order. While most of this research has been based on the Latin alphabet, this is just one of the world’s many writing systems, and there is growing appreciation that “theories and findings cannot be indiscriminately generalized from any one language or writing system to another” (Daniels & Share, 2018, p. 101). At the same time, as all writing systems rely on the visual modality, commonalities may also be expected in letter identification across different scripts. The present study investigates a phenomenon that to date lacks a coherent explanation – namely, stroke neighbor priming effect in Chinese characters – and in doing so, presents evidence to show that the early process of character identification is fundamentally the same in Chinese as that observed with the Latin alphabet and Japanese kana syllabary, likely due to a language-universal visual-perceptual process. We begin with a brief description of the Chinese writing system.

In contrast to alphabetic writing systems where a letter or a letter cluster (e.g., th) maps onto a unit of pronunciation (phoneme), written Chinese is a logographic system in which characters correspond to syllabic morphemes. To attain adult-level literacy, readers of Chinese need to be able to identify more than 3,000 characters. Each (simplified Chinese) character occupies a uniformly-sized square-space and consists of one to 36 strokes that can often be further grouped into small constituents called radicals, which frequently designate the meaning (“semantic radical”) or pronunciation (“phonetic radical”). A large majority (more than 70%) are “compound characters” consisting of two radicals, with the most common arrangement being the semantic radical on the left and the phonetic radical on the right. But there are also sizable “simple characters” that are not decomposable into constituent radicals. Put simply, the Chinese character differs from the most often studied Latin alphabet in at least three respects: It is a logograph, the character inventory is large, and it is visually complex (Yu et al., 2021).^1^

While most (70%) Chinese words comprise two characters, some 20% of words consist of a single character. When only the latter is used as stimuli, “lexical decision” amounts to “character decision.” In this lexical space, an orthographic neighbor is typically defined as sharing a radical (either semantic or phonetic) in the same position. However, defining orthographic neighbors this way likely confounds orthographic similarity with semantic or phonological similarity (Li et al., 2011; Yu & Reichle, 2017). Noting this point, Wang et al. (2014) suggested defining an orthographic neighbor in terms of stroke overlap. This definition also has the advantage that it can apply to simple characters that are not decomposable into constituent radicals.

Many studies investigating the Latin alphabet orthographic processing have used the masked priming procedure with orthographic neighbors. Priming a target with its neighbor has been reported to slow down the identification of the target, especially when the neighboring prime was of higher frequency than the target (e.g., Andrews & Hersch, 2010; Davis & Lupker, 2006). This inhibitory neighbor priming effect is typically explained using the *Interactive-Activation* framework (e.g., Davis, 2003; McClelland & Rumelhart, 1981) that assumes the prior exposure of an orthographic neighbor pre-activates the target word and its lexical competitors, which, in turn, generates lexical competition between the activated words due to lateral inhibition, thereby slowing down the identification of the target word.

In contrast to Latin alphabets, studies investigating masked priming by stroke neighbors in Chinese are scarce, and the observed patterns are mixed. We are aware of only two such studies: Shen and Forster (1999), and Wang et al. (2014). Shen and Forster (1999) used both simple characters (e.g., 目) and compound characters comprised of two radicals (with almost all of them in the left-right configuration, e.g., 侍), and operationally defined the stroke neighbor as a visually similar but phonologically unrelated character that “differed in no more than two strokes from the target” (p. 443). Examples were 日-目, 士-土 (simple characters) and 待-侍 (compound character); for the compound character, the critical stroke could occur either in the semantic radical – as in the example here – or in the phonetic radical. Relative to an unrelated control prime, the stroke neighbor primes produced *facilitatory* priming effects, both in a character-naming task (40 ms and 32 ms for simple and compound characters, respectively) and a lexical decision task (35 ms and 20 ms for simple and compound characters, respectively). The effect of relative prime-target frequency was not investigated.

In contrast, Wang et al. (2014) reported *inhibitory* priming effects by stroke neighbors. Like Shen and Forster (1999), the authors defined a stroke neighbor as a character that differed from its neighbor in one or two strokes, and in Experiments 1 and 2a–2c, used the lexical decision task. The prime duration was varied between experiments: 67 ms in Experiment 1, 33 ms in Experiment 2a, and 50 ms in Experiments 2b and 2c. Wang et al. (2014) found an inhibitory stroke neighbor priming effect (on average ~11 ms) across all four experiments, regardless of whether the prime had a higher or lower frequency than the target. One methodological departure from Shen and Forster’s (1999) masked priming procedure was that in all experiments except Experiment 2c, the prime and target were presented in the same font and in the same size (size 36 Song font). Hence, there was much physical overlap between the stroke neighbor prime and target. Noting this, in their Experiment 2c, Wang et al. presented the targets in Song and primes in Kai font, with primes in a slightly smaller size than the target. Still, the stroke neighbor-priming effect remained inhibitory.

Wang et al. (2014) considered several possible reasons for the discrepancy in the pattern of priming (e.g., differences in the proportion of simple vs. compound characters stimuli), but none provided a satisfactory explanation. One potential factor, not considered by Wang et al., and one that deviates from the predictions of Interactive-Activation frameworks, is the nature of the relationship between stroke neighbor pairs. In both studies, a character’s stroke neighbor was defined as those characters that could be formed mostly by adding or deleting one or more strokes (e.g., 刀 and 刃, 待and 侍);^2^ however, the addition versus deletion stroke neighbors may have different consequences on priming. This prediction is anticipated from the results of recent masked priming letter match studies by Kinoshita et al. (2021) that used visually similar character pairs differing by a visual feature like a stroke.

Kinoshita et al.’s (2021) Experiment 1 used Japanese kana letters with and without diacritics. In the Japanese kana syllabary, the “dakuten” diacritic (which looks like a double apostrophe, placed in the top-right corner above the basic kana) indicates voicing, and turns an unvoiced consonant-initial mora into a voiced one, for example, カ (/ka/) -> ガ (/ga/). This experiment showed that a kana prime without the diacritic (e.g., カ, /ka/) facilitated recognition of a target kana with a diacritic (e.g., ガ, /ga/) almost as much as the identity prime (e.g., ガ–ガ = カ-ガ), whereas a kana prime with the diacritic facilitated recognition of a target kana without a diacritic much less than the identity prime (e.g., カ–カ << ガ–カ). In the present context, the diacriticked prime (e.g., ガ) may be considered as an addition stroke neighbor of the base kana (カ), and conversely, the base kana prime may be considered as a deletion stroke neighbor of its diacriticked counterpart. Kinoshita et al.’s (2021) Experiment 2 used visually similar uppercase letter pairs in the Latin alphabet distinguished by the presence/absence of a single line segment (Q/O, E/F, R/P, G/C). As with the Japanese kana with and without a diacritic, the letter pairs may be regarded as addition or deletion stroke neighbors, respectively. Here too, the pattern of priming was asymmetric such that whereas a deletion stroke neighbor prime facilitated the recognition of the target almost as much as an identity prime (Q-Q = O-Q), an addition stroke neighbor prime produced much less facilitation than an identity prime (O-O << Q-O). Given this consistent pattern of priming asymmetry in two scripts, Kinoshita et al. (2021) explained this asymmetry within the framework of the *Noisy Channel* model (Norris & Kinoshita, 2012). This model assumes that the early stage of letter/word recognition involves the language-universal human visual-object perception system, which is inherently noisy. As is explained fully in the *General discussion*, a relevant property of the visual perception system is that when differentiating stimuli distinguished only by the presence/absence of a feature, an added feature can be more readily detected than a missing feature.

Motivated by this prediction, we investigated whether a similar pattern of priming asymmetry may also be found with the Chinese stroke neighbor characters in a character match task (Experiment 1) and lexical decision task (Experiment 2). A deletion stroke neighbor prime should facilitate the recognition of the target (e.g., 日-目) more than an addition stroke neighbor would (e.g., 目-日) regardless of the task and the relative frequency of the character pairs according to the prediction of Noisy Channel model. We further tested the hypothesis predicted by Interactive-Activation frameworks that any priming effects observed would be modulated by the relative frequency of the stroke neighbor characters, especially in the lexical decision task where the lexical competition is more amplified.

## Experiment 1 (character match task)

### Method

#### Participants

Forty-one undergraduate students (age: 18–22 years; 39 females) from Ludong University, China, took part in this experiment. Given that each condition contained 40 trials, this sample size ensured 1,600 observations in each experimental condition, in line with Brysbaert and Stevens’ (Brysbaert & Stevens, 2018) recommendation for a well-powered masked priming experiment. All participants were native Chinese speakers and had normal or corrected-to-normal vision. Ethics approval was granted by the Psychology Department at Ludong University and written consent was obtained from all participants.

#### Design

The task in Experiment 1 was the masked priming character match task. Each trial involved a referent, prime, and target, presented in that order. Participants were asked to decide if the target was the same character as the referent (no mention was made of the prime). The experimental design involved two factors, Prime type (identity, stroke neighbor) and Target character type (Stroke-present/S+, Stroke-absent/S-), both manipulated within subjects. The dependent variables were response latency and accuracy.

#### Materials

160 characters, arranged as 80 pairs of addition/deletion stroke neighbors, were selected as stimuli. Each pair differed by one stroke (70 pairs) or a fragment of stroke (10 pairs). The character containing the stroke is referred to as Stroke-present (S+), and the character not containing the stroke as Stroke-absent (S-). The critical distinguishing stroke in the S+ character could be attached to (e.g., 自 vs. 目), detached from (e.g., 犬 vs. 大), or intersected with the S- character (e.g., 必vs. 心) (40, 22, and 18 cases, respectively). Note that potentially some characters can be both an S+ or S- character (e.g., the character 白 is an S+ character of character 日, and an S- character of character 百), but, in the experiment, a character was used only once, either as an S+ character or an S- character target. The 80 S+ characters and the 80 S- characters were matched in mean character frequency (1,003 and 716 occurrences per million, *t*(158) = 1.20, *p* > .05), although the relative frequencies of the S- and S+ characters differed for each pair. The full list of stimuli is shown in the Appendix.

The 80 pairs of S+ and S- characters were divided into two half sets matched on mean frequency to counterbalance the assignment of the item to the two prime conditions (identity and stroke neighbor). Each experimental trial consisted of a referent, prime, and target in the character match task. In half of the trials, the referent was the same as the target, and in the other half, the referent was a visually different character from the target, taken from the target character pool, matched on number of strokes and character frequency with the target character. Each character appeared twice as the target in the experiment, once requiring the Same response and once requiring the Different response. The assignment of stimuli to the two prime conditions (identity and stroke neighbor) was counterbalanced using the two half-sets so that each participant saw a given target character once, either with an identity prime or a stroke neighbor prime. Across the two lists, a target occurred with an identity prime and a stroke neighbor prime.

#### Apparatus and procedure

Participants were tested individually, seated approximately 60 cm from the display screen. In Experiment 1, the task was a masked priming character match task. Participants were instructed that they would be presented with two characters in succession (no mention was made of the prime), and their task was to decide whether the two characters were the same. They were instructed to respond as fast as possible without sacrificing accuracy, and press “J” on the keyboard for the Same response, and “F” for the Different response.

Each trial started with the presentation of a fixation cross for 500 ms, followed by a forward mask consisting of 5 # signs and a referent character presented above the mask, for 750 ms. The mask was then replaced by a prime character for 50 ms, followed immediately by a target character. The prime occupied a 150 × 150 pixel space and the target in a 200 × 200 pixel space to avoid physical overlap. All characters were presented in Hei font in black on a white background. Targets were presented for a maximum of 2,000 ms, or until the participant’s response. Participants were given feedback when an error was made.

Each participant completed 320 experimental trials consisting of 160 prime-target pairs (80 S+ targets and 80 S- targets, half paired with an identity prime, and the other half paired with a stroke neighbor prime) presented once with the Same referent and once with a Different referent, over four blocks. Each block contained an equal number of same and different responses, and trials from the four experimental conditions. There was a self-paced break between the blocks, and a different random order of trials was generated for each participant. A practice block of 20 trials preceded the experimental trials.

DMDX software (Forster & Forster, 2003), together with a display screen with a resolution of 1,024 × 1,028 pixels and a refresh rate of 60 Hz, was used to present the stimuli and collect data. Stimulus display was synchronized to the screen refresh rate (~16.7 ms).

#### Analyses

Correct response times (RTs) and accuracy from the Same trials were analyzed using (generalized) linear mixed-effects (LME) models with subjects and items as crossed random factors (Baayen et al., 2008), using the packages lme4 (version 1.1-28; Bates et al., 2018), and lmerTest (version 3.1-3; Kuznetsova et al., 2020) implemented in R (version 3.6.3, 2020-02-29; R Core Team, 2020). Only the RT results are reported here for brevity (results for accuracy resembled the RT data; see Appendix 2). The fixed effects factors were Target character type (S+ or S-) and Prime type (Identity or Stroke neighbor), contrast coded (-.5, .5) to reflect the factorial design. All models started with a maximized random structure with both the intercept and slope included. If a complex model did not converge, or the model fit was not improved by complexity, a simpler model is reported. Emmeans package (version 1.7.2; Lenth, 2022) in R was used to compute the simple effect between the two prime conditions had there been an interaction of the two fixed factors. Finally, the BayesFactor package in R (version 0.9.12-4.3; Morey & Rouder, 2021) was used to quantify the relative strength of evidence for the Target type by Prime type interaction (i.e., priming asymmetry). A Bayes factor is an odds ratio, with 1 indicating equal evidence for the two alternative hypotheses, and, generally, odds of 3 or greater are considered to provide “some evidence,” greater than 10 to be “strong evidence,” and odds greater than 30 to be “very strong evidence” (Dienes, 2014; Jeffreys, 1961).

To examine whether Relative frequency (i.e., character frequency: S- < S+ or S- > S+)^3^ of the stroke neighbor pairs modulates any priming effects observed, we conducted analyses with the Relative frequency of character pairs as an additional fixed factor. One final set of supplementary analyses was also included to test whether there was an effect of how the critical stroke was positioned (i.e., attached, detached, and intersected) relative to the base character. The two Relative frequency levels were contrast coded as (-.5, .5), and simple contrasts were used for the Pair type to compare the differences between the attached versus detached and intersected versus detached character pairs.

### Results

One participant was excluded from data analyses due to the low overall accuracy (61%). The mean correct RT and accuracy for the remaining 40 participants are shown in Table 1.

**Table 1:** Mean response times (in ms) and percent error rate (%E) in Experiment 1 (character match task, “Same” response) and Experiment 2 (lexical decision task, “Word” response)

Target character type	Stroke-absent (e.g., 目)		Stroke-present (e.g., 自)	
Prime type	Example	RT (%E)	Example	RT (%E)
Experiment 1				
Identity	目	527 (2)	自	534 (3)
Stroke neighbor	自	593 (5)	目	536 (3)
Stroke neighbor – Identity		66 (3)		2 (0)
Experiment 2				
Identity	目	607 (4)	自	599 (3)
Stroke neighbor	自	679 (8)	目	618 (5)
Stroke neighbor – Identity		72 (4)		19 (2)

The prime was presented in 150 × 150-pixel space and the target in 200 × 200-pixel space to avoid physical overlap with the prime

*RT* response time

Correct Same-responses trials (*N* = 6,189) were used for the RT analyses, and the RT was log-transformed to meet the distributional assumption of LME. The final model logRT ~ Target character type * Prime type + (1|Subject) + (1|Item) showed that participants responded to S+ targets significantly faster than to S- targets (*t* = -5.74, *p* < .001), and significantly faster when primed by the identity than the stroke-neighbor prime (*t* = 9.56, *p* < .001). Most importantly, the significant interaction (*t* = -8.56, *p* < .001) suggested that the latter effect was only evident for the S- targets (*t* = 12.80, *p* < .001), i.e., priming was asymmetric – a deletion stroke neighbor facilitates target character recognition almost as much as an identity prime, but an addition stroke neighbor interferes with target character recognition relative to an identity prime. The Bayes factor comparing the model including the interaction as the numerator and the model without the interaction as the denominator was 2.96e+14, indicating extremely strong evidence for the interaction.

The final models of logRT ~ Target character type * Prime type * Relative frequency + (1|Subject) + (1|Item) and logRT ~ Target character type * Prime type * Pair type + (1|Subject) + (1|Item) showed no significant effects of Relative frequency (*t* < 1) or Pair type (|*t*| < 1.64, *p* > .05), or three-way interactions (|*t*| < 1.55, *p* > .05), indicating that the stroke neighbor priming asymmetry was not modulated by the relative frequency of the prime-target character pairs, or how the critical distinguishing stroke was positioned relative to the base character. The Bayes factor comparing the models including the three-way interaction as the numerator and the model without as the denominator were 6.92e-04 and 7.10e-06, respectively, providing very strong evidence against the three-way interactions.

## Experiment 2 (lexical decision task)

Experiment 1 used the character-match task that was used in Kinoshita et al. (2021), and extended the asymmetry in stroke neighbor priming effect to Chinese characters. Experiment 2 used the lexical decision task used by Shen and Forster (Shen & Forster, 1999) and Wang et al. (2014) to test whether the priming asymmetry is also found in this task and provide direct comparisons with the priming results reported in these two studies. Furthermore, any modulation of the relative frequency of character pairs on the priming asymmetry is more likely to be observed in the lexical decision task in which frequency is known to play a greater role.

### Method

#### Participants

An additional 40 native Chinese-speaking undergraduate students (age: 18–26 years; 36 females) from Ludong University, China, participated in this experiment for a reward. All had normal or corrected-to-normal vision. Ethics approval was granted by the University’s Psychology Department, and written consent was obtained from all participants.

#### Design

The design was the same as Experiment 1, except that the task was a masked priming lexical decision that required participants to judge whether or not the target was a real character.

#### Materials

The same 80 stroke-neighbor pairs from Experiment 1 and an additional 160 pseudo-characters were used as stimuli. These pseudo-characters were matched with the real characters on the number of strokes, and were constructed by adding or removing stroke(s) to the existing radicals or characters, or reconfiguring the radicals from different characters.

#### Apparatus and procedure

The apparatus and the general procedure were identical to Experiment 1, except participants were instructed to perform a lexical decision task. Each experiment trial started with a 500-ms fixation cross and then a 500-ms forward “#####” mask, followed by a 50-ms prime (occupying a 150 × 150 pixel space), and then a target character displayed in a 200 × 200 pixel space, for 2,000 ms or until the participant’s response. All stimuli were presented on the center screen in Hei font, and participants pressed “J” on the keyboard for Yes responses and “F” for No responses.

#### Analyses

The data analysis method and packages used for the analyses were the same as Experiment 1.

### Results

Participants’ overall accuracy ranged from 87% to 98% (*mean* = 93%, *SD* = 26%), and no participant was excluded from analysis. However, a by-item analysis indicated that six real characters and four pseudo-characters had a lower than 70% accuracy (or, at least 30% of the participants did not judge them correctly). These item pairs were, therefore, excluded from analysis.

Correct real-character trials (*N* = 5,630) were used for the RT analyses, and the RT was log-transformed to meet the distributional assumption of LME. The final model: logRT ~ Target character type * Prime type + (1 + Target character type * Prime type|Subject) + (1|Item) showed that participants responded to S+ targets significantly faster than to S- targets (*t* = -3.19, *p* < .005), and significantly faster when primed by the identity than the stroke-neighbor prime (*t* = 10.16, *p* < .001). Most importantly, the significant interaction (*t* = -2.45, *p* < .05) suggested a priming asymmetry: The identity prime facilitated target recognition more than the stroke neighbor prime for both S- (*t* = 6.09, *p* < .001) and S+ (*t* = 2.29, *p* < .05) targets, but with a larger effect for the former (72 vs. 19 ms, respectively). The Bayes factor analysis provided strong evidence supporting the modeling including the interaction against the model without (*BF*_*10*_ = 2.75e+7).

The final model logRT ~ Target character type * Prime type * Relative frequency + (1 + Target character type * Prime type|Subject) + (1|Item) showed a significant main effect of Relative frequency (*t* = 2.17, *p* < .05). However, no three-way interaction was observed (*t* < 1), indicating that the stroke neighbor priming asymmetry was not modulated by the relative frequency of the prime and target. The Bayes factor analysis similarly showed strong evidence against the three-way interaction (*BF*_*10*_ = 0.03).

The final model logRT ~ Target character type * Prime type * Pair type + (1 + Target character type * Prime type|Subject) + (1|Item) showed no significant main effects of Pair type or three-way interactions (|*t*| < 1.65, *p*s > .05), indicating that the stroke neighbor priming asymmetry was not modulated by how the critical distinguishing stroke was positioned relative to the base character. The Bayes factor analysis provided strong evidence against the three-way interaction (*BF*_*10*_ = 1.48e-3).

## General discussion

The results were straightforward: In both the character match and the lexical decision tasks using single Chinese characters, stroke neighbor primes produced priming effects that were more like the identity prime if the prime was a deletion stroke neighbor of the target (e.g., 目-自>= 自-自) than if it was an addition stroke neighbor (e.g., 自- 目 >> 目-目). This asymmetric pattern of stroke neighbor priming resembled the pattern found with visually similar uppercase letter pairs of the Latin alphabet (e.g., letter pairs Q/O, E/F, R/P, G/C) as well as with the letters of the Japanese kana syllabary with and without a diacritic (e.g., カ/ガ, ス/ズ) (Kinoshita, et al., 2021). Additionally, the relative frequency (i.e., character frequency S- < S+ or S- > S+) and the type (i.e., a stroke in the addition neighbor attached, detached, or intersected with the deletion neighbor) of the stroke neighbor pairs did not modulate this asymmetric pattern of priming effects.

Kinoshita et al. (2021) explained that the priming asymmetry between the deletion and addition stroke neighbors is rooted in the human visual-object perception system (cf. lexical competition) and is anticipated by the Noisy Channel model of word recognition (Norris & Kinoshita, 2012). The Noisy Channel model characterizes orthographic processing in word recognition as a process of recovering a signal being transmitted through a “noisy channel.” A noisy communication channel may corrupt the signal in different ways. For example, it could delete some component of the signal, or it could insert spurious information. To maximize transmission of information through a communication channel, the process responsible for recovering the signal should be optimized to take account of the properties of the channel. In the present context, the relevant property is that a feature is more likely to be lost than added during transmission.

This critical property of the visual object perception system was already known from Treisman and Souther’s (Treisman & Souther, 1985; see also Treisman & Gormican, Treisman & Gormican, 1988) “search asymmetry” experiments. In a series of visual search experiments requiring the detection of a target distinguished from the distractors by a single feature, the authors found that when the target had the feature that was absent in the distractors (e.g., where the distractors are circles and the target is a circle with an intersecting line segment, much like the letters O and Q), the target popped out, whereas when the target lacked the feature that was present in the distractors (e.g., the target O amongst the distractor Q), the search was slow, and serial. From this, it follows that in masked priming a prime that contains a feature absent in the target should interfere more with target recognition than a prime that lacks a feature present in the target, as was observed by Kinoshita et al. (2021) and in the present experiment.

This explanation that the priming asymmetry is likely to have resulted from the language-universal noisy visual-perceptual process is also compatible with the lack of modulation of priming effect by relative prime-target frequency in our study and that reported in Wang et al.’s (Wang et al., 2014). The previously reported inhibitory neighbor priming effect is generally explained in terms of the lexical competition between the target word and the neighbor prime word (e.g., in the Interactive-Activation framework): A higher-frequency neighbor prime (e.g., able-AXLE) “wins” the lexical selection and inhibits the target word’s lexical processing (Davis & Lupker, 2006). The fact that the priming asymmetry was independent of the relative prime-target character frequency is consistent with the early perceptual locus where lexical frequency does not play a role.

We should note that the asymmetry in priming by stroke neighbors here was assessed relative to the identity prime, whereas it is standard to assess priming against an unrelated control prime, as was done by Shen and Forster (Shen & Forster, 1999) and by Wang et al. (2014). However, Kinoshita et al. (Kinoshita et al., 2021, Experiment 1) also included an unrelated control prime, and the priming asymmetry with the “stroke neighbor” prime was observed whether it was assessed against the identity prime or the unrelated control prime. Note also that the asymmetry in the priming effect observed here with the deletion versus addition stroke neighbors was very large (64 ms in the character match task, and 53 ms in the lexical decision task), comparable, if not larger than the difference in the size of stroke neighbor priming effect observed by Shen and Forster (Shen & Forster, 1999, on average 20- to 35-ms facilitatory effect) and Wang et al. (Wang et al., 2014, on average 11-ms inhibitory effect). Thus, we believe the difference in the proportion of deletion and addition stroke neighbors offers a potential explanation for the mixed patterns of priming reported by those researchers.

To conclude, we presented evidence that in Chinese character recognition, deletion stroke neighbor primes facilitate target recognition much more than addition stroke neighbors do. This asymmetry mirrors that observed with the letters of the Latin alphabet and Japanese kana (Kinoshita et al., 2021). This consistency across the writing systems was anticipated by the Noisy Channel model (Norris & Kinoshita, 2012) that views the early stage of letter recognition as essentially the same as recognition of other visual objects. We believe a deeper understanding of reading would be gained by recognizing both the meaningful universals and language/script-specific characteristics of the writing systems.

## Appendix 1

**Table 2:** Full stimulus list

Item number	S- target	S+ target	Item number	S- target	S+ target
1	二	三	41	儿	兀
2	人	个	42	十	千
3	几	凡	43	卜	下
4	刀	刃	44	厂	广
5	刁	习	45	万	方
6	又	叉	46	夕	歹
7	大	犬	47	尸	尺
8	么	公	48	巳	巴
9	之	乏	49	巾	币
10	小	少	50	干	午
11	弓	引	51	口	曰
12	门	闩	52	士	壬
13	勺	匀	53	予	矛
14	王	玉	54	内	丙
15	乌	鸟	55	廿	甘
16	日	旧	56	止	正
17	木	术	57	牛	生
18	从	丛	58	火	灭
19	旦	亘	59	今	令
20	占	乩	60	戊	戌
21	丘	乓	61	业	亚
22	免	兔	62	冉	再
23	丁	于	63	未	朱
24	九	丸	64	白	百
25	了	子	65	目	自
26	川	卅	66	皿	血
27	弋	戈	67	古	舌
28	中	申	68	艮	良
29	云	去	69	西	酉
30	勿	匆	70	乒	兵
31	尤	龙	71	匕	七
32	心	必	72	己	已
33	斤	斥	73	上	土
34	月	用	74	元	无
35	史	吏	75	丰	韦
36	由	曲	76	天	夫
37	幻	幼	77	矢	失
38	囚	因	78	田	甲
39	仝	全	79	石	右
40	哀	衰	80	佘	余

## Appendix 2. Results of accuracy measure

**Experiment 1**

Accuracy data (*N* = 6,400) were analyzed with a generalized linear mixed effects model with subjects and stimuli as crossed random effect factors, using the logit function appropriate for categorical variables (Jaeger, 2008). A final model Accuracy ~ Target character type * Prime type + (1+Prime type|Subject) + (1|Item) showed that accuracy was higher when primed by the identity than stroke neighbor prime (*z* = -2.82, *p* < .005) and a Target character type * Prime type interaction (*z* = 2.13, *p* < .05), with a higher response accuracy for the identity prime than the stroke neighbor prime for the S- targets (*z* = -3.56, *p* < .001) but not for the S+ targets (*z* = -1.00, *p* = .315). The Bayes factor analysis indicated equivocal evidence for the interaction (*BF*_10_ = 0.62).

Consistent with the RT measure, there were no significant effects of Relative frequency, or Pair types, or Target character type * Prime type * Relative frequency/Pair type three-way interactions (|*z*|s < 1.82, *p*s > .05) in accuracy [final models: Accuracy ~ Target character type * Prime type * Relative frequency + (1|Subject) + (1|Item) and Accuracy ~ Target character type * Prime type * Pair type + (1|Subject) + (1|Item)]. The Bayes factor analyses indicated extremely strong evidence against the three-way interactions (*BF*_10_ = 3.85e-04 and *BF*_10_ = 2.14e-06, respectively).

**Experiment 2**

In the analysis of accuracy, 5,920 real-character trials were used, with generalized linear mixed-effects model with subjects and stimuli as crossed random effect factors, using the logit function appropriate for categorical variables. A final model of Accuracy ~ Target character type * Prime type + (1|Subject) + (1|Item) showed participants responded more accurately to the identity than stroke neighbor prime (*z* = -4.38, *p* < .001), more accurately to stroke-present than stroke-absent target (*z* = 2.06, *p* < .05), but the interaction between the two factors was non-significant (*z* = 1.18, *p* > .05). The Bayes factor analysis similarly indicated equivocal evidence for the interaction (*BF*_*10*_ = 0.32).

The final model: Accuracy ~ Target character type * Prime type * Relative frequency + (1|Subject) + (1|Item), showed no main effect of Relative frequency (*z* = -1.26, *p* > .05) but a significant three-way interaction (*z* = -2.47, *p* < .05). However, this significant three-way interaction was inconsistent with the Bayes factor analysis result (*BF*_*10*_ = 0.02), suggesting strong evidence against the three-way interaction.

The final model of Accuracy ~ Target character type * Prime type * Pair type + (1|Subject) + (1|Item) showed no effects of Pair type or three-way interactions (|*z*|s < 1.10, *p*s > .05), and the Bayes factor analyses similarly provided strong evidence against the three-way interactions (*BF*_*10*_ = 8.70e-06).

## Footnotes

1 It is of interest to note that recent analyses of visual complexity across the world’s writing systems have confirmed that the visual complexity of the character is proportional to inventory size (Chang et al., 2018), and that this relationship is driven mainly by a logographic system like Chinese (Miton & Morin, 2021). In other words, the three characteristics of Chinese writing systems “go together.”

2 A small portion of the orthographic neighbors in both Shen and Forster (1999) and Wang et al. (2014) were substitution neighbors, in which the critical stroke(s) occurred in a different spatial position (as in 犬 and 太) or changed its shape (as in 卷 and 券).

3 In 43 stroke neighbor pairs, S- characters had a lower frequency than S+ characters, and in the other 37 pairs, S- characters had a higher frequency than the S+ characters.
